# The Toll of Daily Dehumanization: Elucidating the Role of Dehumanizing Deindividuation by Partner in the Individual and Relational Well‐Being of Women During Pregnancy

**DOI:** 10.1111/famp.70199

**Published:** 2026-09-20

**Authors:** Allison M. Sparpana, Sarah J. Gervais, Rebecca L. Brock

**Affiliations:** ^1^ Department of Psychology The University of Nebraska‐Lincoln Lincoln Nebraska USA

**Keywords:** couples, dehumanization, intimate partner, mood, pregnancy

## Abstract

The purpose of this study was to examine associations between daily experiences of intimate partner dehumanizing deindividuation and pregnant women's emotional and relational well‐being. We also examined uncompassionate self‐responding as a path through which dehumanizing deindividuation exerts its negative effects. A community sample of 154 pregnant women in the United States completed daily surveys over 14 days assessing dehumanizing deindividuation experiences from partner, negative and positive affect, relationship satisfaction, and uncompassionate self‐responding. Dynamic structural equation modeling (DSEM) was used to examine same‐day and lagged associations among these constructs, controlling for prior‐day levels. Same‐day dehumanizing deindividuation was associated with increased negative affect, decreased positive affect, reduced relationship satisfaction, and increased uncompassionate self‐responding. Uncompassionate self‐responding was also associated with more negative affect as well as less positive affect and relationship satisfaction. No next‐day spillover effects were observed. Taken together, dehumanizing treatment from an intimate partner is associated with immediate decrements in emotional and relational functioning, and these experiences appear to undermine individual and relational health directly and potentially through internalization of uncompassionate responses. Interventions aimed at reducing dehumanizing deindividuation and uncompassionate self‐responding should be explored.

## 1 Introduction

Pregnancy is a joyful and exciting transition for many couples, while at the same time it also represents a time of increased stress and vulnerability. Indeed, many pregnant women experience depression and decrements in wellbeing (Biaggi et al. 2016; Pearlstein 2015), which can have downstream effects on offspring (Dunkel Schetter and Tanner 2012). Risk factors for maternal depression include: a history of depression, anxiety, life stress, unintended pregnancy, low income, and lower education; however, dysfunction in the couple relationship (i.e., lack of social support and intimate partner violence) appears to be uniquely harmful (Lancaster et al. 2010). Nonetheless, this research has been largely restricted to one or two relational processes, overlooking other key elements of the couple relationship that could pose significant risk for maternal depression. In addition to escalating depressive symptoms, the pregnancy‐postpartum transition is associated with notable decline in relationship satisfaction (Lawrence et al. 2008, 2010), and there is a need for innovative research identifying relationship processes that contribute to discord in the unique pregnancy context.

One relational process that is emerging as a potential risk factor for decrements in mental health and relational satisfaction—regardless of pregnancy status—is dehumanization by one's intimate partner (Brock and Gervais 2025; Pizzirani et al. 2019). People may assume that closeness and attachment insulate couples from it, but dehumanization in its many forms has been increasingly documented in couples; for example, people can dehumanize their partners by seeing and treating them as sexual objects (Brock et al. 2025), animals (Pizzirani et al. 2019), machines (Pizzirani et al. 2019), or by failing to individuate them (i.e., deindividuation or seeing partner as an extension of oneself and not as a unique individual and placing them outside the circle of mutual care and concern; Brock and Gervais 2025; see also Kelman 1973). Such dehumanizing perceptions and treatment in intimate relationships undermine both relationship and individual functioning. For example, seeing a partner as more animal‐like (e.g., child‐like and unrefined) and more machine‐like (e.g., exploitable and cold) is associated with psychological and physical abuse in the relationship (Pizzirani and Karantzas 2019; Pizzirani et al. 2019), and dehumanization in the form of sexually objectifying behaviors is associated with intimate partner violence alongside depression, body dissatisfaction, and sexual dysfunction (Brock et al. 2025; see also Fredrickson and Roberts 1997).

One dimension of dehumanization that has recently emerged as a predictor of relationship functioning is dehumanizing deindividuation, where one belittles their partner and denies their partner autonomy and uniqueness. Specifically, Brock and Gervais (2025) propose an integrated framework that conceptualizes dehumanizing deindividuation as derogation (e.g., looking down on partner), disregard (e.g., dismissing or ignoring partner), and denial of autonomy (e.g., limiting partner's self‐determination). They found that dehumanizing deindividuation is related to but distinct from measures of other manifestations of dehumanization and individuation in couples, representing a distinct dimension of relationship functioning. Indeed, dehumanizing deindividuation is associated with less emotional intimacy, support, sexual quality, affective communication, and problem solving with their partner and less global relationship satisfaction even when controlling for other risk factors (i.e., individuality in couples; Brock and Gervais 2025).

Dehumanizing deindividuation from a partner may be particularly impactful for women during pregnancy. Pregnancy represents a time of unique dehumanization for women. Due to the salience of their reproductive capacity, society (e.g., through the media, laws, and norms) dehumanizes and reduces pregnant women to reproductive vessels (Dyer et al. 2023; Kahalon and Klein 2025), undermining their autonomy and subjectivity, especially given recent legislative restrictions on reproductive healthcare in the United States (Nolen et al. 2025). Such societal dehumanization could seep into the relational context, where partners may see pregnant women less as individuals, undermining their autonomy and treating them with indifference or contempt (i.e., dehumanizing deindividuation). Although direct evidence is limited, several studies demonstrate that feeling less respected and valued by one's intimate partner during pregnancy can undermine women's emotional health (i.e., mood) and relationship health (i.e., relationship satisfaction). For example, women who feel less humanized by their partners during pregnancy are at risk for depression, eating disorder symptomology, and sexual dissatisfaction (Brock et al. 2021), and lack of mutual respect and acceptance predicts less relationship satisfaction during pregnancy (Ramsdell et al. 2020). Together, these findings are consistent with the possibility that dehumanizing deindividuation from a partner will increase risk for depression and undermine relationship quality during pregnancy, which may be detrimental to couples' abilities to navigate the pregnancy‐postpartum transition, though these associations have yet to be explored.

### 1.1 This Study

Prior research on dehumanizing deindividuation has largely relied on long‐term retrospective self‐reports, which may miss subtle and fleeting behaviors, such as small slights or belittling comments, that may characterize fluctuations in dehumanizing deindividuation in intimate relationships (Brock and Gervais 2025). These low‐level behaviors, though less memorable, may erode same‐day mood or relationship satisfaction. Daily diary methods may offer a stronger approach to capturing shifts in dehumanizing deindividuation and its immediate consequences on mood and relationship satisfaction. In this study, we examined whether daily increases in dehumanizing deindividuation from a partner during pregnancy reduced both emotional well‐being and relationship satisfaction. Specifically, we hypothesized that daily increases in dehumanizing deindividuation would be associated with same‐day mood disturbances associated with depression (i.e., increased negative affect and decreased positive affect; Crawford and Henry 2004; Naragon‐Gainey 2019), and reduced relationship satisfaction (Aim 1). We also explored whether these effects persisted into the next day.

Additionally, there might be an insidious path through which dehumanizing deindividuation undermines individual and relationship health. Specifically, it may be internalized, leading women to view themselves more harshly and as more isolated from others. Thus, we considered uncompassionate self‐responding (Neff and Tóth‐Király 2022), which consists of self‐judgment, experiencing isolation from fellow human beings (a logical, but detrimental outcome of dehumanization), and overidentification with personal shortcomings as a marker of this internalization process. We hypothesized that daily increases in dehumanizing deindividuation by partner would be associated with same‐day increased uncompassionate self‐responding (Aim 2). We also explored whether this effect spilled over into the next day. This internalized perspective could serve as a mechanism through which dehumanizing deindividuation by partner undermines affect and relationship satisfaction. Providing indirect evidence of this possibility, one study found that less humanization was associated with more depression, and seeing the self in a dehumanized manner (i.e., self‐objectification as a sexual object) emerged as an indirect effect explaining this relation (Brock et al. 2021). Further, recent research has demonstrated that uncompassionate self‐responding reflects a key process underlying psychopathology risk (Wadsworth et al. 2018), particularly for depression and anxiety (Muris et al. 2024). In fact, Gerhardt et al. (2024) found that uncompassionate self‐responding, as compared to compassionate self‐responding, was the strongest predictor of postpartum depression. Thus, we hypothesized that daily increases in uncompassionate self‐responding would be associated with same‐day increased negative affect, decreased positive affect, and decreased relationship satisfaction (Aim 3). We also explored whether these effects persisted into the next day.

## 2 Method

### 2.1 Participants and Procedures

One hundred fifty‐nine pregnant women in committed intimate mixed‐sex relationships enrolled in the study. The average age of participants was 28.67 years old (SD = 4.27), and women were mostly in the second (38.4%) or third (58.5%) trimester of pregnancy. Participants reported a median joint income of US$60,000 to US$69,999 and most identified as White and Non‐Hispanic/Latino (83.6%). Average length of women's current intimate relationships was 81.90 months (SD = 49.59) and average length of cohabitation was 61.00 months (SD = 41.80). Most women were married (84.9%). Recruitment was conducted via flyers placed at clinics and businesses visited by pregnant women. All study procedures were approved by the University of Nebraska‐Lincoln Institutional Review Board, No. 20151215700EP. Informed consent was obtained at a laboratory visit during pregnancy, after which participants completed 14 consecutive days of 10–15‐min surveys from home. Payments were prorated based on the number of daily surveys completed for up to US$50. Five of the participants who enrolled in the study ultimately declined participation in the daily surveys; thus, 154 women were included in analyses. Participants completed an average of 12.23 days of surveys (SD = 3.23) across the 14‐day assessment period.

### 2.2 Measures

#### 2.2.1 Dehumanizing Deindividuation by Partner

To assess daily dehumanizing deindividuation by one's intimate partner, we included three items reflecting behaviors that deny a partner's autonomy while derogating and disregarding the partner. These daily items were informed by the conceptualization and scale development of more stable, past‐month relational dynamics reflective of dehumanizing deindividuation (Dehumanizing Deindividuation in Couples scale (DDC); see Brock and Gervais 2025), but were adapted to capture state‐like, daily level fluctuations in such behaviors by partners. Notably, the DDC has demonstrated good convergent validity with other measures of relationship quality (e.g., problem‐solving communication, intimacy, and support) but also assesses a unique facet of the relationship. The DCC has also demonstrated incremental validity, such that DCC scores predicted global relationship satisfaction even when controlling for other relationship processes (e.g., problem‐solving communication, intimacy, and support). Taken together, dehumanizing deindividuation as measured by the DDC (from which the items in the present study were adapted) appears to uniquely predict relationship functioning (i.e., relationship satisfaction and IPV perpetration and victimization; Brock and Gervais 2025). The three daily diary items used in this study were designed to capture several central manifestations of this construct while balancing the practical constraints of repeated daily, state‐like assessment (e.g., reducing participant burden). Accordingly, the daily diary measure should be viewed as an abbreviated, state assessment of key features of dehumanizing deindividuation rather than a comprehensive measure of the broader construct. Specifically, participants were asked to indicate how much they agreed with the following statements: (a) “My partner treated me like a child rather than like an equal partner in the relationship”, (b) “My partner suggested that there was something s/he would like to change about me”, and (c) “My partner belittled me for or made spiteful comments about my hobbies, career, habits, passions, etc.” Participants responded to each item using a 1 (*strongly disagree*) to 5 (*strongly agree*) scale, and items were averaged for composite scores ranging from 1 to 5. Internal consistency was excellent (*α* = 0.84). See the Results Section 3 for estimates of internal consistency at separate within‐ and between‐person levels in the tested multilevel models.

#### 2.2.2 Daily Mood

We used the brief version of the Positive and Negative Affect Schedule (PANAS‐X; Watson and Clark 1994). Participants were asked to rate the extent to which they experienced 10 negative emotions (e.g., afraid, hostile, guilty, ashamed, and upset) and 10 positive emotions (e.g., alert, enthusiastic, excited, inspired, and strong) that day on a scale ranging from 1 (*very slightly or not at all*) to 5 (*extremely*). Items were summed for scores ranging from 10 to 50 for negative affect and 10 to 50 for positive affect. Internal consistency for the negative affect (*α* = 0.88) and positive affect (*α* = 0.96) scales were excellent.

#### 2.2.3 Daily Relationship Satisfaction

The Quality of Marriage Index (QMI; Norton 1983) is a 6‐item, self‐report questionnaire. Items were modified to refer to the “relationship with my partner” rather than “marriage.” Participants endorsed the extent to which they agreed or disagreed with five items (e.g., “our relationship is strong”) using a scale from 1 (*very strong disagreement*) to 7 (*very strong agreement*) and rated their global relationship happiness on a scale from 1 (*very unhappy*) to 10 (*perfectly happy*). Items were summed with higher scores (ranging from 6 to 45) suggesting greater satisfaction. Internal consistency was excellent (*α* = 0.95).

#### 2.2.4 Uncompassionate Self‐Responding

We used three subscales from the short form version of the Self‐Compassion Scale (SCS‐SF; Neff 2003) that represent uncompassionate self‐responding including self‐judgment (e.g., being intolerant of one's own flaws; *α* = 0.88), isolation (e.g., feeling alone in failures; *α* = 0.74), and overidentification (e.g., being consumed with feelings of inadequacy; *α* = 0.74). Questions on the SCS are rated on a 5‐point scale ranging from 1 (*almost never*) to 5 (*almost always*). Subscale scores (mean of 2 items) demonstrated large intercorrelations (*r*s ranged from 0.61 to 0.70) and were averaged to obtain an internally consistent composite of uncompassionate self‐responding with scores ranging from 1 to 5.

### 2.3 Data Analysis

We implemented dynamic structural equation modeling (DSEM; Asparouhov et al. 2018) which disaggregates variance into within‐person (time‐varying) and between‐person (time‐invariant) counterparts. Analyses were conducted in Mplus 8.2 using a Markov chain Monte Carlo (MCMC) procedure with default priors, 2 MCMC chains, and the Gibbs algorithm. This is a full‐information approach, so all cases were retained for analysis despite missing data (covariance coverage ranged from 0.927 to 0.930). We used the default potential scale reduction (PSR) convergence criteria which concludes convergence when PSR nears 1.0. We also doubled the number of iterations to ensure that the PSR value remained near 1.0. We examined the 95% credibility intervals (CI), based on posterior distributions, for each of the hypothesized effects. If the CI did not contain zero, the effect was deemed significant. We modeled daily dehumanizing deindividuation by partner as a predictor of same‐day and next‐day reports of mood and relationship satisfaction (Aim 1) and same‐day and next‐day reports of uncompassionate self‐responding (Aim 2). We modeled daily uncompassionate self‐responding as a predictor of same‐day and next‐day reports of mood and satisfaction (Aim 3). We controlled for previous day levels of variables, and all effects were modeled as random. Data management and general analysis procedures for this study were preregistered (https://osf.io/hprk8), but given our prior knowledge of data from this longitudinal study, we did not preregister study hypotheses.

## 3 Results

### 3.1 Correlations and Variance Across Levels

Correlations at the within‐person and between‐person levels are reported in Table 1. All constructs demonstrated adequate reliability at both levels (i.e., dehumanizing deindividuation *ω*ᵂ = 0.77; *ω*ᴮ = 0.92; negative affect *ω*ᵂ = 0.87, *ω*ᴮ = 0.94; positive affect *ω*ᵂ = 0.93, *ω*ᴮ = 0.98; relationship satisfaction *ω*ᵂ = 0.93, *ω*ᴮ = 0.98; and uncompassionate self‐responding *ω*ᵂ = 0.73, *ω*ᴮ = 0.97). Dehumanizing deindividuation had an ICC of 0.36 suggesting that 64% of the variance in those scores was at the daily level. ICCs were 0.44 for negative affect, 0.61 for positive affect, 0.57 for relationship satisfaction, and 0.76 for uncompassionate self‐responding. Although study aims were focused on associations at the within‐person level, it was notable that there were significant correlations, in expected directions, among all variables at the between‐person level (small to moderate in size).

**TABLE 1:** Correlations and descriptive statistics.

Variable	1	2	3	4	5
1. Negative affect	—	−0.31***	−0.30***	0.20***	0.30***
2. Positive affect	−0.32***	—	0.26***	−0.16***	−0.23***
3. Relationship satisfaction	−0.11	0.36***	—	−0.36***	−0.18***
4. Dehumanizing deindividuation	0.30**	−0.28***	−0.66***	—	0.20***
5. Uncompassionate self‐responding	0.57***	−0.19**	−0.19*	0.36***	—
*M*	13.12	34.10	41.53	1.29	2.00
SD	4.84	9.21	5.59	0.61	0.91

*Note:* Correlations at the within‐person level are reported above the diagonal, and correlations at the between‐person level are reported below the diagonal.

* *p* < 0.05.

** *p* < 0.01.

*** *p* < 0.001.

### 3.2 Aim 1: Same‐Day and Next‐Day Associations Between Dehumanizing Deindividuation and Mood and Relational Outcomes

Controlling for previous day levels of variables, daily increases in dehumanizing deindividuation were associated with same‐day increases in negative affect, *b* = 1.91, 95% CI [1.36, 2.44], *β* = 0.26, *τ* = 3.44 (between‐subject variability in effect), and decreases in positive affect, *b* = −2.15, 95% CI [−2.80, −1.52], *β* = −0.18, *τ* = 1.91, and relationship satisfaction, *b* = −2.93, 95% CI [−3.67, −2.28], *β* = −0.35, *τ* = 7.12. Autoregressive effects were significant and small in magnitude (*β* = 0.25 for negative affect, *β* = 0.26 for positive affect, *β* = 0.29 for relationship satisfaction). This model explained 30% of the within‐person variance in negative affect, 25% of positive affect, and 46% of satisfaction. Dehumanizing deindividuation was not associated with any of the outcomes on the next day (*b* = 0.33 for negative affect, *b* = −0.39 for positive affect, and *b* = −0.44 for satisfaction).

### 3.3 Aim 2: Same‐Day and Next‐Day Associations Between Dehumanizing Deindividuation and Uncompassionate Self‐Responding

Controlling for previous day levels of variables, increases in dehumanizing deindividuation were associated with same‐day increases in uncompassionate self‐responding, *b* = 0.21, 95% CI [0.10, 0.34], *β* = 0.20, *τ* = 0.16. This model explained 34% of the within‐person variance in uncompassionate self‐responding. The autoregressive effect was significant and small in magnitude (*β* = 0.29 for uncompassionate self‐responding). Dehumanizing deindividuation was not associated with next‐day uncompassionate self‐responding, *b* = 0.00, 95% CI [−0.09, 0.08], *β* = 0.02, *τ* = 0.04.

### 3.4 Aim 3: Same‐Day and Next‐Day Associations Between Uncompassionate Self‐Responding and Outcomes

Controlling for previous day levels of variables, increases in uncompassionate self‐responding were associated with same‐day increases in negative affect, *b* = 2.15, 95% CI [1.56, 2.71], *β* = 0.30, *τ* = 6.66 and decreases in positive affect, *b* = −2.92, 95% CI [−3.52, −2.25], *β* = −0.24, *τ* = 1.91 and satisfaction, *b* = −1.30, 95% CI [−1.85, −0.76], *β* = −0.17, *τ* = 4.64. The autoregressive effects were significant and small to moderate in magnitude (*β* = 0.23 for negative affect, *β* = 0.25 for positive affect, *β* = 0.36 for relationship satisfaction, *β* = 0.35 for uncompassionate self‐responding). This model explained 35% of the within‐person variance in negative affect, 28% of positive affect, and 37% of satisfaction. Uncompassionate self‐responding was not associated with next‐day levels of the outcomes (*b* = 0.20 for negative affect, *b* = −0.71 for positive affect, and *b* = −0.17 for satisfaction). Note that given uncompassionate self‐responding did not predict next‐day outcomes, and mood and relationship satisfaction is expected to reciprocally influence uncompassionate self‐responding, we did not formally test for mediation pathways (e.g., dehumanizing deindividuation ➔ uncompassionate self‐responding ➔ negative affect) given insufficient basis for causal inference.

## 4 Discussion

Consistent with hypotheses connected to Aim 1, results suggest that when pregnant women experience daily increases in dehumanizing deindividuation by their intimate partner (Brock and Gervais 2025; e.g., being treated as a child or belittled), this is associated with daily increases in negative affect and decreases in positive affect and relationship satisfaction, controlling for previous day levels. In particular, there was a moderate effect size for relationship satisfaction, relative to small effects for affect. Further, it was notable that a large portion (nearly two‐thirds) of the variance in scores of dehumanizing deindividuation was at the daily level, suggesting that women's experiences of dehumanizing deindividuation by one's partner can fluctuate day‐to‐day during pregnancy with consequences for both individual and relational well‐being. These findings are consistent with dehumanization theories (Fredrickson and Roberts 1997; Haslam 2006) and recent research suggesting that dehumanization occurs in intimate relationships with negative consequences, including depression (Brock et al. 2021) and relationship dissatisfaction (Brock and Gervais 2025; Sáez et al. 2019), but extends this work revealing that dehumanizing deindividuation has negative effects on both relational as well as individual functioning. The same‐day effects did not persist into the next day, pointing to a time‐limited effect.

Results of Aim 2 were consistent with our hypothesis that one potential process through which daily dehumanizing deindividuation impacts the well‐being of pregnant women is increased uncompassionate self‐responding (i.e., self‐judgmental thoughts about one's own flaws and inadequacies, feeling alone in one's inadequacies, and overidentification with feelings of inadequacy). Indeed, theories and related research on dehumanization (Baldissarri et al. 2022; Brock et al. 2021; Fredrickson and Roberts 1997; Haslam 2006) suggest that a particularly insidious consequence of dehumanization is internalized dehumanization or self‐dehumanization. For example, research reveals that being reduced to a sexual object by one's partner increases self‐objectification (Brock et al. 2021; Fredrickson and Roberts 1997). The present results point to a related internalization process, namely uncompassionate self‐responding; specifically, dehumanizing deindividuation from an intimate partner that undermined pregnant women's individuality was associated with internalized uncompassionate self‐responding within the same day. However, this effect did not appear to persist into the next day, suggesting the effects of dehumanizing deindividuation on self‐responding might be limited to the same day.

Aim 3 results were consistent with hypotheses and provide further evidence that uncompassionate self‐responding might function as a mechanism through which daily dehumanizing deindividuation undermines individual and relational well‐being. To the extent that women reported higher levels of uncompassionate self‐responding on a given day, they also reported more negative affect and less positive affect and relationship satisfaction on the same day, controlling for previous day levels. The effect was most pronounced for negative affect, which had a moderate effect size relative to small effects for positive affect and relationship satisfaction. Thus, the extent to which women internalize messages of dehumanizing deindividuation from their partners during pregnancy—manifested in the form of self‐judgment, isolation from humanity, and overidentification—might have serious implications for well‐being, especially negative affect and corresponding risk for developing mood and anxiety disorders (Crawford and Henry 2004; Naragon‐Gainey 2019). This is particularly relevant given substantial empirical evidence demonstrating increased risk for developing depression and comorbid anxiety disorders during pregnancy and postpartum (Biaggi and Pariante 2020). These results also converge with research suggesting that negative aspects of self‐compassion have close ties with psychopathology, particularly depression and anxiety (Muris et al. 2024). However, uncompassionate self‐responding was only associated with individual and relational outcomes on the same day, not on the next day, and it is possible that mood disturbances and relationship dissatisfaction could exacerbate uncompassionate self‐responding; therefore, the directionality of this association remains unclear and might ultimately reflect a reciprocal process. Accordingly, we did not formally test for mediation (e.g., dehumanizing deindividuation ➔ uncompassionate self‐responding ➔ negative mood), but further consideration of uncompassionate self‐responding as a mechanism explaining the negative consequences of dehumanizing deindividuation for individual and relational functioning is warranted in future research.

### 4.1 Theoretical, Methodological, and Clinical Implications

There are several important implications of this work. First, dehumanizing deindividuation by an intimate partner during pregnancy may be a particularly salient risk factor for reduced wellbeing, given that pregnancy is already marked by threats to personhood and autonomy from society and others (e.g., medical professionals, strangers, and acquaintances). Pregnant women are often portrayed as reproductive vessels whose wellbeing is less valuable than the fetus (Dyer et al. 2023; Kahalon and Klein 2025), face reductions in autonomy through legislative restrictions (Nolen et al. 2025) and obstetric violence (Kahalon and Klein 2025), and encounter public scrutiny of their daily activities (e.g., Sutton et al. 2011). Our results suggest that when these societal dynamics surface within intimate relationships, they have detrimental consequences to individual and relational health.

By capturing dehumanizing deindividuation in intimate relationships on a daily basis, our results offer a more nuanced view of how subtle, often‐overlooked behaviors (e.g., belittling comments and covert autonomy denial) shape well‐being. Long‐term accounts may obscure these fluctuations, but we found that day‐to‐day elevations in partner dehumanizing deindividuation are linked to more negative affect and uncompassionate self‐responding and less positive affect and relationship satisfaction. Significant between‐person associations also suggest that more stable tendencies in dehumanizing deindividuation relate to maternal well‐being. Thus, there may be advantages to measuring both state‐like and more stable tendencies of dehumanizing deindividuation. Such understanding may inform more precise intervention targets. Future research is needed to determine whether these findings generalize beyond pregnancy to other relationship stages and contexts (e.g., dating, coparenting of young children, and retirement).

Our results also identify uncompassionate self‐responding as a potential mechanism through which pregnant women internalize dehumanizing treatment from their partner, which then negatively impacts well‐being. Existing frameworks linking dehumanization in couples to detrimental outcomes have largely overlooked mechanisms through which these effects unfold (e.g., Pizzirani et al. 2019; c.f., Brock et al. 2025). Although spillover effects were absent, reciprocal processes are possible; uncompassionate self‐responding may impact individual and relational well‐being, but negative (and less positive) mood and relationship dissatisfaction may also exacerbate uncompassionate self‐responding. Taken together, the present study identifies uncompassionate self‐responding as a potential mechanism through which dehumanizing deindividuation affects individual and relational well‐being during pregnancy, yet future research is needed to disentangle the directionality of these effects.

Clinically, our results point to the need to address dehumanizing deindividuation by intimate partners in perinatal interventions. Even low‐level acts of dehumanizing deindividuation may accumulate, eroding well‐being and relationship quality. Interventions that bolster respectful communication and emotional intimacy (e.g., Emotionally Focused Therapy for Couples; Wiebe and Johnson 2016; see Kanter and Schramm 2018 for a review of brief couples interventions that may be more easily implemented during the perinatal period) may buffer against these effects. Attention to relational power dynamics, often shaped by larger societal attitudes toward pregnant women (Dyer et al. 2023; Kahalon and Klein 2025), may also be critical, given that power differentials have been found to lead to dehumanizing others (Gwinn et al. 2013). Clinicians may target such dynamics by fostering attunement between partners and encouraging the vulnerability of the powerful partner (Knudson‐Martin et al. 2015). Finally, given that uncompassionate self‐responding may be a mechanism through which dehumanizing deindividuation is internalized by pregnant women and affects individual and relational health, interventions such as Acceptance and Commitment Therapy and Mindfulness‐Based Stress Reduction (Wilson et al. 2019) may protect against maladaptive outcomes of dehumanizing deindividuation from partner.

### 4.2 Limitations and Future Directions

There were some limitations of this study. First, as expected in a community sample, pregnant women reported relatively low levels of dehumanizing deindividuation, negative affect, and uncompassionate self‐responding and relatively high positive affect and relationship satisfaction. Research should examine the tested associations in pregnant women experiencing more dysfunctional relational dynamics and clinical levels of emotional disorders. Second, the sample consisted of mixed‐sex couples and was predominantly White, which limits the generalizability of findings. Women from minoritized backgrounds are at significantly greater risk for psychopathology during the perinatal period (e.g., Gavin et al. 2011; Robinson et al. 2016), experience higher rates of verbal and physical abuse during pregnancy (e.g., D'Angelo et al. 2022; O'Campo et al. 1994), and are at an elevated risk for pregnancy complications (e.g., Bornstein et al. 2020; Everett et al. 2021; Guendelman et al. 2006; Minehart et al. 2021). Finally, although the daily diary items captured core features of dehumanizing deindividuation, future research would benefit from more comprehensive daily measures of the broader construct and examinations of the incremental prediction of dehumanizing deindividuation when controlling for other daily relationship processes. Thus, it is imperative that research closely examine risk factors for psychopathology and relationship discord (including intimate partner violence) in diverse populations.

## 5 Conclusion

In conclusion, the daily experience of dehumanizing treatment from an intimate partner during pregnancy was associated with same‐day declines in emotional and relational well‐being. Specifically, women's perceived dehumanizing deindividuation by their partner predicted higher negative affect, lower positive affect, reduced relationship satisfaction, and heightened uncompassionate self‐responding. Further, daily uncompassionate self‐responding was associated with increased negative affect, decreased positive affect, and lower relationship satisfaction. These associations emerged within the same day, but did not carry over to the subsequent day, suggesting that the harmful effects of daily dehumanizing deindividuation may be acute. Together, these findings are some of the first to highlight dehumanizing deindividuation as an important relationship process connected to mental and relationship health for women during pregnancy and point to uncompassionate self‐responding as one pathway explaining these connections.

## Funding

This research was funded by several internal funding mechanisms awarded to PI Rebecca Brock from the UNL Department of Psychology, the Nebraska Tobacco Settlement Biomedical Research Development Fund, and the UNL Office of Research and Economic Development.

## Ethics Statement

All study procedures were approved by the University of Nebraska‐Lincoln (UNL) Institutional Review Board, Family Development Project (#20151215700EP).

## Consent

Informed consent was obtained from all study participants.

## Conflicts of Interest

The authors declare no conflicts of interest.

## Data Availability

This study complied with Transparency and Openness Promotion (TOP) Guidelines. The study PI, Rebecca L. Brock (rebecca.brock@unl.edu), should be contacted to request access to research materials, analysis code, and data. Data management and general data analysis procedures for this project were preregistered at https://osf.io/hprk8, and we made no deviations from that plan. Because we had prior knowledge of data from this longitudinal study, we did not preregister our hypotheses. Data from this sample have been published elsewhere (e.g., Brock et al. 2021; Grandgenett et al. 2024); however, this is the first article in which daily deindividuation by partner was linked to daily individual and relational well‐being in pregnant women.

## References

[famp70199-bib-0001] Asparouhov, T. , E. L. Hamaker , and B. Muthén . 2018. “Dynamic Structural Equation Models.” Structural Equation Modeling: A Multidisciplinary Journal 25, no. 3: 359–388. 10.1080/10705511.2017.1406803.

[famp70199-bib-0002] Baldissarri, C. , S. Demoulin , and N. Kteily . 2022. “Introduction to the Special Issue of Group Processes & Intergroup Relations Less Than Human: What People Who Are Dehumanized Think, Feel, and Do.” Group Processes & Intergroup Relations 25, no. 8: 1927–1938. 10.1177/13684302221139414.

[famp70199-bib-0004] Biaggi, A. , S. Conroy , S. Pawlby , and C. M. Pariante . 2016. “Identifying the Women at Risk of Antenatal Anxiety and Depression: A Systematic Review.” Journal of Affective Disorders 191: 62–77. 10.1016/j.jad.2015.11.014. PMC4879174 26650969

[famp70199-bib-0047] Biaggi, A. , and C. M. Pariante . 2020. “Risk Factors for Depression and Anxiety During the Perinatal Period.” In Handbook of Perinatal Clinical Psychology, edited by R. M. Quatraro and P. Grussu , 1st ed., 239–265. Routledge. 10.4324/9780429351990-18.

[famp70199-bib-0005] Bornstein, E. , Y. Eliner , F. A. Chervenak , and A. Grünebaum . 2020. “Racial Disparity in Pregnancy Risks and Complications in the US: Temporal Changes During 2007–2018.” Journal of Clinical Medicine 9, no. 5: 1414. 10.3390/jcm9051414. PMC7290488 32397663

[famp70199-bib-0007] Brock, R. L. , and S. J. Gervais . 2025. “The Psychometric Properties of a New Scale of Dehumanizing Deindividuation in Couples.” Journal of Family Psychology 39, no. 2: 171–183. 10.1037/fam0001311. 39847006

[famp70199-bib-0008] Brock, R. L. , S. J. Gervais , O. R. Checkalski , S. D. Finkelstein , and A. M. Sparpana . 2025. “A Multidimensional Measure of Sexual Objectification in Intimate Relationships: The Inventory of Partner Sexual Objectification (IPSO).” Psychological Assessment 37: 720–734. 10.1037/pas0001411. 40742767

[famp70199-bib-0009] Brock, R. L. , E. L. Ramsdell , G. Sáez , and S. J. Gervais . 2021. “Perceived Humanization by Intimate Partners During Pregnancy Is Associated With Fewer Depressive Symptoms, Less Body Dissatisfaction, and Greater Sexual Satisfaction Through Reduced Self‐Objectification.” Sex Roles 84, no. 5–6: 285–298. 10.1007/s11199-020-01166-6.

[famp70199-bib-0010] Crawford, J. R. , and J. D. Henry . 2004. “The Positive and Negative Affect Schedule (PANAS): Construct Validity, Measurement Properties and Normative Data in a Large Non‐Clinical Sample.” British Journal of Clinical Psychology 43, no. 3: 245–265. 10.1348/0144665031752934. 15333231

[famp70199-bib-0011] D'Angelo, D. V. , J. M. Bombard , R. D. Lee , K. Kortsmit , M. Kapaya , and A. Fasula . 2022. “Prevalence of Experiencing Physical, Emotional, and Sexual Violence by a Current Intimate Partner During Pregnancy: Population‐Based Estimates From the Pregnancy Risk Assessment Monitoring System.” Journal of Family Violence 38, no. 1: 117–126. 10.1007/s10896-022-00356-y. PMC10193455 37205924

[famp70199-bib-0012] Dunkel Schetter, C. , and L. Tanner . 2012. “Anxiety, Depression and Stress in Pregnancy: Implications for Mothers, Children, Research, and Practice.” Current Opinion in Psychiatry 25, no. 2: 141–148. 10.1097/YCO.0b013e3283503680. PMC4447112 22262028

[famp70199-bib-0013] Dyer, R. L. , O. R. Checkalski , and S. J. Gervais . 2023. “Abortion Decisions as Humanizing Acts: The Application of Ambivalent Sexism and Objectification to Women‐Centered Anti‐Abortion Rhetoric.” Psychology of Women Quarterly 47, no. 4: 528–546. 10.1177/03616843231173673.

[famp70199-bib-0014] Everett, B. G. , A. Limburg , B. M. Charlton , J. M. Downing , and P. A. Matthews . 2021. “Sexual Identity and Birth Outcomes: A Focus on the Moderating Role of Race‐Ethnicity.” Journal of Health and Social Behavior 62, no. 2: 183–201. 10.1177/0022146521997811. PMC10368195 33687305

[famp70199-bib-0015] Fredrickson, B. L. , and T.‐A. Roberts . 1997. “Objectification Theory: Toward Understanding Women's Lived Experiences and Mental Health Risks.” Psychology of Women Quarterly 21, no. 2: 173–206. 10.1111/j.1471-6402.1997.tb00108.x.

[famp70199-bib-0016] Gavin, A. R. , J. L. Melville , T. Rue , Y. Guo , K. T. Dina , and W. J. Katon . 2011. “Racial Differences in the Prevalence of Antenatal Depression.” General Hospital Psychiatry 33, no. 2: 87–93. 10.1016/j.genhosppsych.2010.11.012. PMC3880676 21596200

[famp70199-bib-0017] Gerhardt, B. C. , J. G. Serra , C. Zimmer , and A. X. Arteche . 2024. “Role of Self‐Criticism in Postpartum Mental Health: A Network Analysis.” Psicologia: Reflexão e Crítica 37, no. 1: 38. 10.1186/s41155-024-00321-2. PMC11401827 39276252

[famp70199-bib-0048] Grandgenett, H. M. , M. R. Franz , F. C. Calkins , D. DiLillo , and R. L. Brock . 2024. “Does Past Intimate Partner Violence Moderate the Link Between Daily Stress and Conflict During Pregnancy? A Dyadic Daily Diary Investigation.” Psychology of Violence 14, no. 4: 239–249. 10.1037/vio0000515.

[famp70199-bib-0018] Guendelman, S. , D. Thornton , J. Gould , and N. Hosang . 2006. “Obstetric Complications During Labor and Delivery: Assessing Ethnic Differences in California.” Women's Health Issues 16, no. 4: 189–197. 10.1016/j.whi.2005.12.004. 16920523

[famp70199-bib-0019] Gwinn, J. D. , C. M. Judd , and B. Park . 2013. “Less Power = Less Human? Effects of Power Differentials on Dehumanization.” Journal of Experimental Social Psychology 49, no. 3: 464–470. 10.1016/j.jesp.2013.01.005.

[famp70199-bib-0020] Haslam, N. 2006. “Dehumanization: An Integrative Review.” Personality and Social Psychology Review 10, no. 3: 252–264. 10.1207/s15327957pspr1003_4. 16859440

[famp70199-bib-0021] Kahalon, R. , and V. Klein . 2025. “Unmasking the Role of Dehumanization in Obstetric Violence.” Psychology of Violence 15, no. 1: 21–31. 10.1037/vio0000521.

[famp70199-bib-0022] Kanter, J. B. , and D. G. Schramm . 2018. “Brief Interventions for Couples: An Integrative Review.” Family Relations 67, no. 2: 211–226. 10.1111/fare.12298.

[famp70199-bib-0023] Kelman, H. G. 1973. “Violence Without Moral Restraint: Reflections on the Dehumanization of Victims and Victimizers.” Journal of Social Issues 29, no. 4: 25–61. 10.1111/j.1540-4560.1973.tb00102.x.

[famp70199-bib-0024] Knudson‐Martin, C. , D. Huenergardt , K. Lafontant , L. Bishop , J. Schaepper , and M. Wells . 2015. “Competencies for Addressing Gender and Power in Couple Therapy: A Socio Emotional Approach.” Journal of Marital and Family Therapy 41, no. 2: 205–220. 10.1111/jmft.12068. 24844561

[famp70199-bib-0025] Lancaster, C. A. , K. J. Gold , H. A. Flynn , H. Yoo , S. M. Marcus , and M. M. Davis . 2010. “Risk Factors for Depressive Symptoms During Pregnancy: A Systematic Review.” American Journal of Obstetrics and Gynecology 202, no. 1: 5–14. 10.1016/j.ajog.2009.09.007. PMC2919747 20096252

[famp70199-bib-0026] Lawrence, E. , A. D. Rothman , R. J. Cobb , and T. N. Bradbury . 2010. “Marital Satisfaction Across the Transition to Parenthood: Three Eras of Research.” In Strengthening Couple Relationships for Optimal Child Development: Lessons From Research and Intervention, edited by M. S. Schulz , M. K. Pruett , P. K. Kerig , and R. D. Parke , 97–114. American Psychological Association. 10.1037/12058-007.

[famp70199-bib-0027] Lawrence, E. , A. D. Rothman , R. J. Cobb , M. T. Rothman , and T. N. Bradbury . 2008. “Marital Satisfaction Across the Transition to Parenthood.” Journal of Family Psychology 22, no. 1: 41–50. 10.1037/0893-3200.22.1.41. PMC2367106 18266531

[famp70199-bib-0028] Minehart, R. D. , A. S. Bryant , J. Jackson , and J. L. Daly . 2021. “Racial/Ethnic Inequities in Pregnancy‐Related Morbidity and Mortality.” Obstetrics and Gynecology Clinics of North America 48, no. 1: 31–51. 10.1016/j.ogc.2020.11.005. 33573789

[famp70199-bib-0029] Muris, P. , I. Fernández‐Martínez , and H. Otgaar . 2024. “On the Edge of Psychopathology: Strong Relations Between Reversed Self‐Compassion and Symptoms of Anxiety and Depression in Young People.” Clinical Child and Family Psychology Review 27, no. 2: 407–423. 10.1007/s10567-024-00471-w. PMC11222199 38472504

[famp70199-bib-0030] Naragon‐Gainey, K. 2019. “Affective Models of Depression and Anxiety: Extension to Within‐Person Processes in Daily Life.” Journal of Affective Disorders 243: 241–248. 10.1016/j.jad.2018.09.061. 30248635

[famp70199-bib-0031] Neff, K. D. 2003. “The Development and Validation of a Scale to Measure Self‐Compassion.” Self and Identity 2, no. 3: 223–250. 10.1080/15298860309027.

[famp70199-bib-0032] Neff, K. D. , and I. Tóth‐Király . 2022. “Self‐Compassion Scale (SCS).” In Handbook of Assessment in Mindfulness Research, edited by O. N. Medvedev , C. U. Krägeloh , R. J. Siegert , and N. N. Singh , 1–22. Springer International Publishing. 10.1007/978-3-030-77644-2_36-1.

[famp70199-bib-0033] Nolen, E. , K. Cary , R. R. Mendoza , S. Vohra‐Gupta , and C. Cubbin . 2025. ““It's More Important Than Ever for Men to Play a Role”: Women's Perspectives on Safe Sex Responsibilities Following the Dobbs Decision.” Journal of Sex Research 63: 1–10. 10.1080/00224499.2025.2499564. 40378196

[famp70199-bib-0034] Norton, R. 1983. “Measuring Marital Quality: A Critical Look at the Dependent Variable.” Journal of Marriage and the Family 45, no. 1: 141. 10.2307/351302.

[famp70199-bib-0035] O'Campo, P. , A. C. Gielen , R. R. Faden , and N. Kass . 1994. “Verbal Abuse and Physical Violence Among a Cohort of Low‐Income Pregnant Women.” Women's Health Issues 4, no. 1: 29–37. 10.1016/S1049-3867(05)80107-0. 8186724

[famp70199-bib-0036] Pearlstein, T. 2015. “Depression During Pregnancy.” Best Practice & Research. Clinical Obstetrics & Gynaecology 29, no. 5: 754–764. 10.1016/j.bpobgyn.2015.04.004. 25976080

[famp70199-bib-0037] Pizzirani, B. , and G. C. Karantzas . 2019. “The Association Between Dehumanization and Intimate Partner Abuse.” Journal of Social and Personal Relationships 36, no. 5: 1527–1541. 10.1177/0265407518811673.

[famp70199-bib-0038] Pizzirani, B. , G. C. Karantzas , and E. R. Mullins . 2019. “The Development and Validation of a Dehumanization Measure Within Romantic Relationships.” Frontiers in Psychology 10: 2754. 10.3389/fpsyg.2019.02754. PMC6908499 31866918

[famp70199-bib-0039] Ramsdell, E. L. , M. Franz , and R. L. Brock . 2020. “A Multifaceted and Dyadic Examination of Intimate Relationship Quality During Pregnancy: Implications for Global Relationship Satisfaction.” Family Process 59, no. 2: 556–570. 10.1111/famp.12424. 30615199

[famp70199-bib-0040] Robinson, A. M. , K. M. Benzies , S. L. Cairns , T. Fung , and S. C. Tough . 2016. “Who Is Distressed? A Comparison of Psychosocial Stress in Pregnancy Across Seven Ethnicities.” BMC Pregnancy and Childbirth 16, no. 1: 215. 10.1186/s12884-016-1015-8. PMC4982239 27514674

[famp70199-bib-0041] Sáez, G. , A. R. Riemer , R. L. Brock , and S. J. Gervais . 2019. “Objectification in Heterosexual Romantic Relationships: Examining Relationship Satisfaction of Female Objectification Recipients and Male Objectifying Perpetrators.” Sex Roles 81, no. 5–6: 370–384. 10.1007/s11199-018-0990-9.

[famp70199-bib-0042] Sutton, R. M. , K. M. Douglas , and L. M. McClellan . 2011. “Benevolent Sexism, Perceived Health Risks, and the Inclination to Restrict Pregnant Women's Freedoms.” Sex Roles 65, no. 7–8: 596–605. 10.1007/s11199-010-9869-0.

[famp70199-bib-0043] Wadsworth, L. P. , M. Forgeard , K. J. Hsu , S. Kertz , M. Treadway , and T. Björgvinsson . 2018. “Examining the Role of Repetitive Negative Thinking in Relations Between Positive and Negative Aspects of Self‐Compassion and Symptom Improvement During Intensive Treatment.” Cognitive Therapy and Research 42, no. 3: 236–249. 10.1007/s10608-017-9887-0.

[famp70199-bib-0044] Watson, D. , and L. A. Clark . 1994. “The PANAS‐X: Manual for the Positive and Negative Affect Schedule – Expanded Form.” [Dataset]. 10.17077/48vt-m4t2.

[famp70199-bib-0045] Wiebe, S. A. , and S. M. Johnson . 2016. “A Review of the Research in Emotionally Focused Therapy for Couples.” Family Process 55, no. 3: 390–407. 10.1111/famp.12229. 27273169

[famp70199-bib-0046] Wilson, A. C. , K. Mackintosh , K. Power , and S. W. Y. Chan . 2019. “Effectiveness of Self‐Compassion Related Therapies: A Systematic Review and Meta‐Analysis.” Mindfulness 10, no. 6: 979–995. 10.1007/s12671-018-1037-6.

